# The Role of Gut Microbiota in Aging and Aging Related Neurodegenerative Disorders: Insights from *Drosophila* Model

**DOI:** 10.3390/life11080855

**Published:** 2021-08-20

**Authors:** Yan Kong, Liyuan Wang, Baichun Jiang

**Affiliations:** 1 Department of Biochemistry and Molecular Biology, School of Medicine, Southeast University, Nanjing 210009, China; 220193631@seu.edu.cn; 2 The Key Laboratory of Experimental Teratology, Ministry of Education, Department of Molecular Medicine and Genetics, School of Basic Medical Sciences, Shandong University, Jinan 250012, China; jiangbaichun@sdu.edu.cn

**Keywords:** aging, neurodegeneration, *Drosophila*, microbiota, Alzheimer’s disease, Parkinson’s disease

## Abstract

Aging is characterized by a time dependent impairment of physiological function and increased susceptibility to death. It is the major risk factor for neurodegeneration. Neurodegenerative disorders including Alzheimer’s disease (AD) and Parkinson’s disease (PD) are the main causes of dementia in the old population. Gut microbiota is a community of microorganisms colonized in the gastrointestinal (GI) tract. The alteration of gut microbiota has been proved to be associated with aging and aging related neurodegeneration. *Drosophila* is a powerful tool to study microbiota-mediated physiological and pathological functions. Here, we summarize the recent advances using *Drosophila* as model organisms to clarify the molecular mechanisms and develop a therapeutic method targeting microbiota in aging and aging-related neurodegenerative disorders.

## 1. Introduction

Aging is characterized by a time dependent impairment of physiological function and increased susceptibility to death [1]. It is the major risk factor for a plethora of human diseases, including cancer, metabolic syndrome, cardiovascular disorders, and neurodegeneration. Neurodegenerative disorders, including Alzheimer’s disease (AD) and Parkinson’s disease (PD), are the main causes of dementia in the elderly [2]. Gut microbiota is a community of microorganisms colonized in the gastrointestinal (GI) tract, including bacteria, viruses, protozoa, and fungi [3]. The gut microbiota contributes to development, metabolic homeostasis and physiology [4]. Dysbiosis is defined as imbalance of microbial communities in the GI tract and associates with aging and aging related neurodegeneration [5].

With short lifespan and easy genetic manipulation, *Drosophila* is recognized as a powerful tool to clarify molecular mechanism of development and diseases. Many mammalian tissues including the heart and kidney have equivalent parts in *Drosophila*, which is absent in *C. elegans*, another widely used model organism in aging research. It has been found that two thirds of human disease associated genes and all major signaling pathways are conserved in *Drosophila* [6]. Insulin/IGF-1 like signaling and mechanistic Target of Rapamycin (mTOR) signaling, key pathways control *Drosophila* longevity, have been proved to mediate aging process in mammals [7].

The *Drosophila* microbial community is composed of 5–20 different microbial species, including the *Lactobacillus*, *Acetobacter*, *Enterococcus*, and *Leuconostoc*, which is much less complex than mammals [8]. Besides, Germ free (GF) or gnotobiotic *Drosophila* is much easier to obtain and culture in large quantities. Findings from *Drosophila* studies on microbiota–host interaction have potential for translating into mammals. For example, gut microbiota promotes juvenile growth conservatively in *Drosophila* and mice [9,10]. Monocolonized GF flies and mice with *Lactobacillus plantarum*^WJL^ recapitulate the beneficial effects of the microbiota on postnatal growth [10]. These advantages make *Drosophila* an ideal model organism to study microbiota-mediated physiological functions in aging and molecular pathology of neurodegenerative disorders.

## 2. Microbiota in Aging *Drosophila*

### 2.1. Microbiota in Human Aging

The microbial organisms colonize in the GI tract after birth and *Bifidobacterium* genus is dominant for infants [11]. The diversity increases after the first year. The adult microbiota is dominated by *Firmicutes*, *Bacteroides*, *Proteobacteria* and *Actinobacteria* [12]. Aging reduces the biodiversity of human gut microbiota and the abundance of *Bifidobacteria*, *Lactobacilli*, and short chain free fatty acids (SCFAs) producers, such as *Faecalibacterium prausnitzii*, *Eubacterium* spp., *Roseburia* spp., and *Ruminococcus* spp. [13]. Studies carried out in China and Italy separately report that microbial community richness contributes to longevity [14,15]. The shared alteration of microbiota with aging could be found in *Blautia*, *Clostridium cluster XIVa*, *Faecalibacterium*, *Escherichia_Shigella*, *Lachnospiraceae*, *Ruminococcaceae*, and *Erysipelotrichaceae*. A study on three independent cohorts comprising over 9000 individuals shows that depletion of core genera, primarily *Bacteroides*, is beneficial for healthy aging [16]. The gut microbiota of long-living people (aged over 90 years) has also been investigated. By metagenomic sequencing of stool samples, healthy long-living people have a higher abundance of *Bacteroidetes* and several functional metabolic pathways [17]. In contrast, the unhealthy long-living group contains a higher abundance of *Streptococcus* and more functional pathways for xenobiotics biodegradation. These reports from human studies are not very consistent due to differences in race, lifestyle and diet. It requires further investigations using various model organisms to clarify the role of microbiota in aging.

### 2.2. Alteration of Microbiota in Drosophila Aging

*Proteobacteria* (*Acetobacter* and *Komagataeibacter*) and *Firmicutes* (*Lactobacillus* and *Leuconostoc*) comprise the major part of the gut microbiota in *Drosophila* [18]. The stomach-like copper cell region (CCR) in the middle midgut controls distribution and composition of the microbiota [19]. The abundance and richness of the microbial species increase dramatically with age. *Acetobacter persici* and *Lactobacillus brevis* are dominant species in young flies while *Acetobacter malorum* and *Lactobacillus plantarum* are dominant in old *Drosophila* [20]. Axenic flies could be obtained by embryo bleaching and culture on sterile food. The second and third generation of axenic flies live much longer than those raised conventionally. This observation is reproduced in antibiotics treated conventionally cultured flies. Further experiments show that increased microbial abundance is a stronger determinant of host lifespan than microbial composition [20,21].

When germ free (GF) adult *Drosophila* were inoculated with a cocktail of bacterial species, including *Acetobacter pomorum*, *Lactobacillus plantarum*, *Enterococcus faecalis*, *Acetobacter* sp., and *Leuconostoc pseudomesenteroides*, aging shifts the gut microbiota composition as indicated by β-Diversity and PCoA assay [22]. Absolute quantification of the total bacterial cell number by qPCR shows that bacterial load increases with age. Dietary restriction (DR) during adulthood has an evolutionary conserved anti-aging effect [23]. When subjected to a regime of 2 day fed and 5 day fasted for one month, *Drosophila* lifespan increases significantly. The load of bacteria decreases, and the gut barrier function is improved at day 40 post intermittent fasting treatment.

The effects of microbial organisms on *Drosophila* aging are not very consistent, which could be explained by several reasons. The time-point for *Drosophila* to be colonized with microbial organisms is important for their effects on longevity. There is a lifespan enhancing effect for bacteria when it is delivered to axenic *Drosophila* during the first week of adult life [24]. However, contact with bacteria in later life reduces the survival time significantly [24]. The nutritional environment should also be considered when analyzing the role of microbiota on aging. The lifespan of *Drosophila* could either be extended or shortened by microbes when they are cultured under malnourishing (low yeast) diets or rich (high yeast) diets [25]. Axenic *Drosophila* could be obtained either by egg bleaching or antibiotic treatment, which may cause toxic effects and affect the lifespan. Lee et al. report that the lifespan of axenic flies obtained by sodium hypochlorite-based bleaching method is much shorter than conventionally reared flies [20]. However, the 2nd and 3rd generations of axenic flies after 1st generation bleaching live much longer than conventionally reared flies. Detrimental effect could also be observed in antibiotics treated axenic *Drosophila*. The diluted antibiotic cocktail with no toxic effects on axenic *Drosophila* prolongs the lifespan of conventionally reared flies [20].

### 2.3. Mechanisms of Microbiota in Drosophila Aging

Gene ontology (GO) analysis identifies aging related major categories of gene expression in flies are immunity, olfaction/sensation, stress response, rhythmic behavior, and metabolism. The alteration of 70% of aging induced genes, especially for stress-resistance (Hsp70, Hsp26, and Hsp27) and activation of innate immunity (CecC, DptB, and AttA), disappears in axenic *Drosophila* cultured on media with antibiotics for multi-generations [21]. Accordingly, axenic flies demonstrate more resistance to oxidative stress, starvation and challenge to *Drosophila* pathogen *Erwinia carotovora subsp. carotovora* (ATCC 15390). In contrast, the processes of rhythmic behavior, chitin-based cuticle development, and sensory perception of smell are still enriched in aged axenic-raised flies. In addition to Toll signaling pathway, the *Drosophila* immune response is regulated by the immune deficiency (IMD) pathway, which controls the expression of several antimicrobial peptides (AMPs). Aging increases the expression of several AMPs, such as diptericin, drosocin and attacin A, in *Drosophila* gut and whole-body. These effects could be abolished by add back of *A. aceti* at adulthood, suggesting that microbiome alteration could drive IMD hyperactivation during aging. Metabolic pathways participating in the aging process are also found to be influenced by gut microbiota. Allantoin, an end product of purine metabolism, is increased during aging. *Acetobacter persici* in *Drosophila* gut could activate IMD pathway in the renal tubules and promotes allantoin production [26].

Metagenome-wide association (MGWA) identifies cysteine and methionine metabolism contributing to longevity in *Drosophila* [27]. Flies inoculated with *Acetobacter fabarum* ectopically expressing cystathionine beta synthase (CBS), which drives flux through transsulfuration and restricts methionine content, demonstrate improved longevity. Compared with lifespan, healthspan is the length of time that an individual remains healthy without neurodegeneration and other aging related disorders. Microbiota derived indoles extend the healthspan of various organisms [28]. Germ free *Drosophila* raised with K12 *E. coli* has improved the lifespan, climbing ability and resistance to heat stress, which are abolished after the mutation of indole synthesis essential gene tryptophanase (tnaA). The aryl hydrocarbon receptor (AHR) is the direct receptor for indoles. K12 *E. coli* could not improve the healthspan in AHR mutant flies, indicating the critical role of AHR in the molecular mechanism for indole in healthy aging. These findings from *Drosophila* are conserved in *C. elegans* and mice, further proving *Drosophila* as a powerful tool in the research of microbiota in aging.

Gut is the first barrier for microbiota and their bidirectional interaction contributes to the aging process. Alteration in the microbiota activates immune genes and precedes intestinal barrier dysfunction in aged *Drosophila* [29]. Following intestinal barrier disruption, microbiota composition alters dramatically and induces systemic immune activation. Lactate, produced by *L. plantarum* in the gut microbiota, could be oxidized into pyruvate and releases NADH in the enterocyte. The NADPH oxidase Nox utilizes NADH to produce ROS and promotes intestinal stem cells (ISCs) proliferation. Subsequent gut hyperplasia will shorten the lifespan in aged flies. Host immune dysfunction can also lead to dysbiosis that in turn promotes aging. Constitutive activation of the gut immune system by mutation of Pdm1/nubbin (nub), a POU transcriptional regulator, increased the abundance of bacteria and richness of microbiota composition [30]. The lifespan shortening effects of nub mutation were abolished after antibiotics treatment. Peptidoglycan recognition proteins (PGRPs) are innate immunity molecules conserved from insects to mammals. Mutation of PGRP in *Drosophila* leads to overgrowth of *Lactobacillus plantarum* and lactic acid production, which promotes Nox mediated ROS production, intestinal damage, intestinal stem cell proliferation, and dysplasia [29]. Activation of JAK/Stat signaling in the gut induces age-related metaplasia, commensal dysbiosis and gut functional decline, which ultimately decreases the lifespan of *Drosophila* [19].

The epigenetic mechanism also contributes to the role of microbiota in aging [31]. Epigenetic modifications contain DNA methylation, histone modification (acetylation and methylation) and non-coding RNAs. The histone demethylase KDM5 is responsible for histone H3K4me3 demethylation and regulates gene expression of transcriptionally active genes [32]. Whole body or gut specific disruption of KDM5 damages intestinal epithelial barrier and decreases the number of species in the gut microbiota. KDM5 mutant flies demonstrate a much shorter lifespan [33]. KDM5 mutation in *Drosophila* increases the level of *Proteobacteria* and decreases the level of Firmicutes. At the orders level, *Sphingomonadales*, *Enterobacteriales*, and *Xanthomonadales* are dominant while *Lactobacillales*, *Bacteroidales*, and *Bifidobacteriales* are less abundant in KDM5 mutant flies. Antibiotic treatment augments while probiotic treatment with *L. plantarum* L168 rescues these phenotypes. RNA sequencing reveals a critical role of KDM5 in activation of IMD pathway and production of downstream factors, including diptericin A (DptA), attacin-B (AttB), and the RE isoform of peptidoglycan recognition protein LC (PGRP-LC-RE) [32]. These studies give evidence that microbiota may be regulated and work together with epigenetic mechanisms in the aging process.

### 2.4. Antiaging Therapy Targeting Microbiota in Drosophila

In parallel with the research on molecular mechanisms for microbiota in aging, scientists are devoted to identifying antiaging drugs targeting microbiota using *Drosophila* model (Table 1). Carrageenan oligosaccharide (CAO), derived from marine red algae, effectively improved the longevity, motility behavior and fecundity by increasing the diversity of gut microbiota and the abundance of *Commensalibacter* in male *Drosophila* [34]. Agar oligosaccharide (AOS) is a marine prebiotic that promotes longevity and health. In *Drosophila* model, AOS not only significantly activates intestinal immune related IMD pathway but also augments the lifespan and resistance to oxidative stress [35]. AOS significantly decreases the diversity of microbiota in aged *Drosophila*. Among the dominant microbes in the gut, the abundance of *Gluconobacter* increases from 28.99% to 69.18% while *Lactobacillus* and *Acetobacter* are much lower. Aqueous furbelow extracts (brown algae, *Saccorhiza polyschides*) could also increase species richness of microbiota and extend the lifespan, especially in high-fat and drought diet [36]. Glucomannan hydrolysates (GMH) are derived from the root of the *Amorphophallus konjac* plant. It is found that GMH extends the lifespan and increases the abundance rather than diversity of *Drosophila* gut microbiota [37]. Inulin is a fermentable dietary fiber contained in fruits, vegetables, and herbs. Inulin prolongs the lifespan only in male flies by impacting the gut microbiota. *Lactobacillaceae* is dominant in male flies on the standard diet, while *Streptococcaceae* is enriched in males fed with inulin [38].

In addition to saccharides, ursolic acid (UA), a natural triterpenoid, is beneficial to the climbing ability and lifespan of males *Drosophila* [39]. UA affected the bacterial composition and population distribution of microbiota. *Actinobacteria* is the phyla demonstrating significant difference in abundance after UA supplementation. Improved lifespan and locomotion are abolished when *Drosophila* is raised in axenic conditions, confirming that the antiaging effects of UA rely on gut microbiota. The same observations are also found for lithocholic acid (LCA), the secondary bile acid generated by gut microbiota from primary bile acids. Antibiotics inhibit its beneficial effects on lifespan extension [40].

“What doesn’t kill you makes you stronger.” Low doses of oxidants treatment at larval stage, including tert-butyl hydroperoxide (tBH) and paraquat, increases the lifespan of *Drosophila* [41]. Mechanism study reveals that they decrease the *A. aceti*, *Komagataeibacter rhaeticus*, *Acetobacteraceae* rather than *Lactobacillus* species in the gut. G418, an antibiotic targeting *A. aceti* and enriching *L. plantarum*, also promotes the longevity. The beneficial effects could be further transferred to the next generation. Further experiments show that *A. aceti* activates the IMD pathway and gut dysfunction during aging, which is abolished by tBDH treatment.

In addition to chemical treatment, physical factors such as visible light also influence aging. Treatment with 12 h light and 12 h dark by color-specific LED at the intensity of 600 lx and 100 lx, green light (550 ± 68 nm) rather than blue light (457 ± 30 nm) or red light (675 ± 75 nm) extends the lifespan of *Drosophila* [42]. Doxycycline (DOX), a chemical that inhibits bacterial load and diversity, abolished the antiaging effects of green light. In the future, noninvasive therapy based on physical stimulation could possibly be developed for aging and aging related disorders.

Although research into antiaging intervention has been progressing rapidly, it should be noted that several studies only demonstrate alteration of diversity and/or abundance of microbiota in parallel with lifespan extension after treatment [34,35,36,37,38]. Further experiments using axenic culture, antibiotics treatment and reintroduction of microbial populations should be performed to clarify whether the antiaging effects of these therapeutic methods rely on targeting microbiota.

## 3. Gut Microbiota in *Drosophila* AD Model

### 3.1. Microbiota in Human AD

Alzheimer’s disease (AD) is the most prevalent reason for dementia in the old population [43]. The pathological features of AD include amyloid plaque, hyperphosphorylation of tau protein and neuronal loss [44]. Tau is a microtubule associated protein and could be hyperphosphorylated in AD patients, which leads to its aggregation into tangles. Amyloid β (Aβ) is derived from sequential processing of APP by BACE and γ secretases. Overproduction or inadequate clearance of Aβ leads to senile plaque formation. Mutations of APP and PS1/2 are usually found in early onset familial Alzheimer’s disease (FAD). However, as most AD cases are sporadic and late onset, the etiology is still elusive.

It is reported that 85% of dementia patients have alterations in gut microbiota [45]. The diversity of gut microbiota is decreased significantly in AD patients [46]. The abundance of *Bacteroides*, *Lachnospiraceae, E. rectale Butyrivibrio*/*Eubacterium*/*Clostridium Firmicutes* and *Bifidobacterium* is decreased while the load of *Ruminococcus*, *Actinobacteria*, *Escherichia/Shigella*, *O. splanchnicus*, *Bacteroidetes* increases significantly [46,47,48,49]. Among them, *Escherichia Shigella*, *Odoribacter splanchnicus*, and *Klebsiella pneumonia* have been proved to be associated with inflammatory state while *Butyrivibrio* and *Eubacterium* exert anti-inflammation effects. Increased prevalence of *Bacteroides* is associated with mild cognitive impairment (MCI) in patients without dementia [50].

Several *Drosophila* models have been established in order to clarify the underlying mechanisms for AD, including elav-Gal4;UAS-BACE/UAS-APP model, elav-Gal4;UAS-Aβ_42_ model and GMR-Aβ_42_ model, which facilitates the research on the contribution of microbiota to AD pathogenesis.

### 3.2. elav-Gal4;UAS-BACE/UAS-APP Model

The elav-Gal4 line pan-neuronally expresses the driver protein Gal4 under the promoter of the elav gene. When they cross with UAS-BACE/UAS-APP line, the resulting F1 flies with the genotype elav-Gal4;UAS-BACE/UAS-APP produce Aβ in the brain and demonstrates neurodegeneration phenotypes. Kefir is a natural probiotic drink constituted by *Lactobacillus kefiranofaciens* (21.96%), *Lactobacillus kefiri* (0.2%), *Acetobacter fabarum* (0.17%), *Lactococcus lactis* (0.004%) and *Rickettsiales* (0.001%) [51]. Kefir treated AD like *Drosophila* demonstrates improved lifespan and climbing ability as compared with water or milk treated group. Liquid–liquid partitioning separates Kefir metabolites into four fractions with increasing polarity: exane (Hex), dichloromethane (DCM), ethyl acetate (EtOAc) and n-butanol (But-OH). All the fractions improve the climbing ability and AD-like vacuolar lesions while EtOAc (0.5 mg/mL) and ButOH (0.5 mg/mL) fractions extend the lifespan of AD like *Drosophila*. GC–MS analysis identifies 117 compounds shared by all fractions, including short-chain fatty acids (SCFAs) which are downregulated in AD *Drosophila* and mice.

Synbiotic formulation could be obtained by combination of probiotic formulation (*Lactobacillus plantarum* NCIMB 8826 (Lp8826), *L. fermentum* NCIMB 5221 (Lf5221) and *Bifidobacteria longum* spp. *infantis* NCIMB 702255 (Bi702255)) and with 0.5% of TFLA (polyphenol plant extract from the gastrointestinal tonic Triphala) powder [52]. Synbiotic treatment ameliorates neurodegeneration as measured by survivability, motility, Aβ accumulation and acetylcholinesterase (ACh) activity fly heads. Mechanism study reveals that synbiotic treatment decreases the expression of *Drosophila* insulin-like peptide (dilp)2, dilp3, InR and upregulates downstream transcription factor dFOXO in insulin signaling of AD flies. The upregulation of innate immune factor dual oxidase, IMD and IMD downstream factors (cytokine-like immune mediator Relish, Attacin A, Diptercin, Defensin) is abolished in AD flies by synbiotic feeding. Synbiotic treatment also reduces the level of total oxidants, lipid peroxidation (LPO) and rescues the activity of the ETC complexes. The beneficial effects of synbiotic feeding relies on PPARγ as proved by bisphenol A diglycidyl ether (BADGE), a PPARγ antagonist treatment.

### 3.3. GMR-Aβ_42_ Model

When UAS-Aβ_42_ virgins are crossed with glass multimer reporter-Gal4 (GMR-Gal4) males, the offspring will demonstrate rough eye phenotype as neurodegeneration. It is widely used in screening assay for AD associated mechanisms. Eye malformation of GMR-Aβ_42_ flies could be reversed prevalently by *Lactobacillus sakei* Probio65, *Lactobacillus paracasei* 0291 and *Lactobacillus plantarum* DR7 (DR7), accompanied with reduced the abundance of *Wolbachia* and increased abundance of *Stenotrophomonas* and *Acetobacter* in gut microbiota [53,54]. PICRUSt analysis, a tool to construct predicted functional metagenomes, shows that *Wolbachia* is positively correlated with neurodegeneration, such as Parkinson’s, Huntington’s and Alzheimer’s diseases, while *Stenotrophomonas* and *Acetobacter* have the opposite effects.

### 3.4. elav-Gal4;UAS-Aβ_42_ Model

AD like *Drosophila* models could also be established by directly expressing Aβ_42_ in pan-neuronal manner. Our group has found that the diversity of microbiota increased dramatically with Aβ_42_ overexpression [55]. As the dominant bacteria in the gut, the proportions of *Acetobacteraceae* and *Lactobacillacea* at the family level while *Acetobacter* and *Lactobacillus* at the genus level decrease dramatically in AD *Drosophila*. GC–MS reveals acetate is the most abundant SCFA and decreases dramatically in AD group. Consistently, the level of SCFAs including acetate decreases significantly in fecal samples from pre-onset amnestic mild cognitive impairment (aMCI) and is further reduced more dramatically in AD patients [56]. Nagpal et al. report slightly decreased fecal acetate and propionate in mild cognitive impairment (MCI) patients [57]. Intragastric administration acetate rescues cognitive impairments and microglia activation in AD (APP/PS1) mice [58]. It should be noted that another study finds that there is no significant difference of acetate and propionate while decreased butyrate in the fecal samples AD mice [59]. SCFAs have also been reported to be reduced in GF AD (APP/PS1) mice. SCFA supplementation increases Aβ plaque, plaque associated microglia recruitment and less intracellular Aβ in microglia [60]. Further experiments are required to clarify the role of SCFAs and other microbiota metabolites in AD pathogenesis.

Enteric dysbiosis could be induced by oral infection with a nonpathogenic *enterobacteria* (Ecc15) in adult flies [61]. The dysbiosis augments AD like phenotypes in *Drosophila* expression Aβ_42_ in the brain without affecting gut barrier, including declined lifespan, climbing ability and increased neuronal loss. Enteric infection promotes the upregulation of the *Drosophila* TNF eiger and downstream JNK activity as well as the production of AMPs (Dpt, Drs, AttA, and CecA1) and ROS. The ROS induced recruitment of plasmatocytes, functional macrophages in *Drosophila*, is increased in the brain of AD flies and triggers TNF-JNK pathway after enteric dysbiosis. This work further highlights the essential role of microbiota mediated gut–brain crosstalk in AD pathogenesis.

## 4. Gut Microbiota in *Drosophila* PD Model

### 4.1. Microbiota in Human PD

Parkinson’s disease (PD) is the second most prevalent neurodegenerative disorder affecting the elderly population [62]. Its predominant pathological features are death of dopaminergic (DA) neurons in the substantia nigra pars compacta and intraneuronal accumulations of Lewy bodies.

The gut microbiota is altered in PD patients [63]. The abundance of *Prevotellaceae*, *Blautia*, *Coprococcus*, *Roseburia*, *Faecalibacterium*, and *Prevotella* decreased while *Enterobacteriaceae*, *E. coli*, *Ralstonia*, *Lactobacillus*, *Bifidobacteriu*, *Verrucomicrobiaceae*, *Bacteroides*, *Parabacteroides*, *Akkermansia*, *Butyricimonas*, *Veillonella*, *Odoribacter*, *Mucispirillum*, and *Bilophila* increase in the gut microbiota of PD patients [63,64,65,66,67]. Subsequent increase of gut permeability could also be found [64]. Among the decreased microbial organisms, the genera of *Blautia*, *Coprococcus*, and *Roseburia* could produce anti-inflammatory butyrate [65]. The abundance of *Bacteroides* is decreased in PD patients with tremors compared to those without this symptom, indicating that the severity of PD correlates with microbiota alteration [67]. SCFAs are significantly downregulated in the gut of PD patients, exerting profound effects on inflammation and gut barrier damage in PD progression [68].

### 4.2. elav- Gal4;UAS-Synuclein Model

As the main component of Lewy bodies, α-synuclein contributes to PD by aggregation into insoluble filaments. Multiplication or mutation (A53T, A30P or E46K) of α-synuclein is found in familial forms of PD patients [62]. The virgin elavC155-GAL4 line is crossed to UAS-α-synucleinA53T males to make the F1 offspring express A53T α-synuclein in the brain as PD model. Treatment with phenolic acid metabolites, including 3-HBA, 3,4-diHBA and 3-HPPA, inhibits the formation of α-synuclein dimers and trimers *in vitro* and improves the climbing ability of PD flies *in vivo* [69]. *B. ovatus* was able to convert flavanols catechin and epicatechin (C/EC) into DHCA, 3,4-diHBA, and 3-HBA. Additionally, *B. ovatus*, *E. lenta*, and *E. coli* are also able to generate DHCA, 3-HPPA, 3,4-diHBA, and 3-HBA through a C/EC-independent process. This study reveals that gut microbiota potentially modulates dietary flavanols to protect against PD pathogenesis.

### 4.3. PINK1 Mutant Model

Mitochondria function related genes, including the Parkin, DJ-1 and PTEN induced putative kinase 1 (PINK1), are identified as PD associated genes. PINK1 is a nucleus encoded gene and is targeted to mitochondria. Animal models with PINK mutation demonstrate fragmented mitochondrial cristae, sensitive to oxidative stress, accompanied with locomotion defects and DA neuron loss. *Drosophila* PINK1 mutants (PINK1^B9^) demonstrate reduced lifespan, climbing and flight defects, degenerated flight muscle, and loss of DA neurons in the PPL1 region, which could be rescued by EGCG supplement [70]. The decreased microbiota diversity is also rescued by EGCG in PINK1^B9^ flies. EGCG decreases *Proteobacteria* and increases *Firmicutes* and *Bacteroidetes* at the phylum level in PD flies. As the dominant genus in *Drosophila* microbiota, the abundance of *Acetobacter* and *Lactobacillus* is inhibited after EGCG treatment in PINK1^B9^ flies. Gut microbial alteration induced by *Lactobacillus plantarum* KJ01 strain blunts the EGCG-mediated rescue effect on the fly locomotion in genetic PD model (PINK1^B9^ flies) and genetic × environmental model (rotenone-exposed PINK1^B9^ flies). This study proposes the key function of microbiota in the neuroprotective role for EGCG in PD.

## 5. Conclusions and Future Perspective

It should be noted that most of the studies mentioned above were performed either in female [20,22,27,32,33,37,39,55,69] or male [21,24,25,26,28,29,34,35,41,52,53,61,70] models. It has been found that the abundance of *A. pasteurianus*, *L. plantarum*, and *L. fructivorans* alters similarly both in aged male and female *Drosophila* [71]. However, both *w^1118^* and canton S female flies harbor more *Enterococcus*, which may interfere with colonization of *Acetobacter* and *Lactobacillus* during aging. Accordingly, female flies live much longer than males both in low yeast and high yeast medium [72]. Males also demonstrate more aging dependent DA neuron loss and locomotion defects [73]. Further investigation is required to validate whether microbiota contribute to the sex-dependent difference in aging and neurodegeneration.

**Figure 1:**
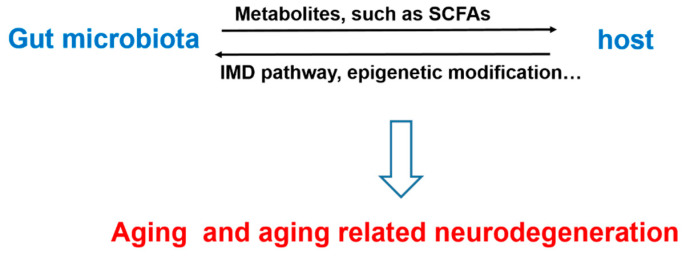
The interaction of gut microbiota and host in aging and aging related neurodegeneration.

With the advantages of simple microbial community composition, *Drosophila* is a powerful tool to clarify the contribution of microbiota to aging and aging related neurodegeneration. Aging induced alteration of microbiota prior to gut damage, which could act on various signaling pathways via metabolites and exert beneficial or detrimental effects on longevity and neurodegeneration (Figure 1). Intervention strategies targeting *Drosophila* microbiota either by chemical or physical treatment have been developed in order to improve healthy aging. Many findings from *Drosophila* have been proved to be highly conserved in mammals, further validating the value of this model in aging and neurodegeneration related microbiota research.

## Figures and Tables

**Table 1:** Antiaging therapy targeting microbiota in *Drosophila*.

Category	Treatment
Chemical	Carrageenan oligosaccharide Agar oligosaccharide Aqueous furbelow extracts Glucomannan hydrolysates Inulin Ursolic acid Lithocholic acid tert-butyl hydroperoxide (low dose) Paraquat (low dose)
Physical	Green light (550 ± 68 nm)

## Data Availability

Not applicable.
